# How can WhatsApp® facilitate the future of medical education and clinical practice?

**DOI:** 10.1186/s12909-020-02440-7

**Published:** 2021-01-14

**Authors:** Muhammed Aizaz us Salam, George Chukwuemeka Oyekwe, Sami Ahmad Ghani, Regwaan Imtiaz Choudhury

**Affiliations:** St George’s University of London, Cranmer Terrace, Tooting, London, SW17 0RE UK

**Keywords:** WhatsApp, Instant messaging application, Communication, Medical education, Problem based learning, Clinical attachment, National Health Service

## Abstract

As part of the modern generation of medical students and prospective future doctors of the United Kingdom’s Nation Health Service (NHS), we have grown up in an age where smartphones and instant messaging applications (IMAs) are ubiquitous across all aspects of society. With IMAs being so familiar, we recognise their scope for facilitating our learning of the pre-registration syllabus and how their practical nature could potentially revolutionise healthcare worldwide. It is, therefore, rational to further investigate the benefits of incorporating such technology into these respective settings. In this article, we will further expand on some of the advantages highlighted by E. Colman & E. O’Connor that IMAs, particularly WhatsApp, have in the academic environment which resonate with us. We illustrate our views on IMAs being incorporated into health systems globally through exemplifying the NHS, using reviewed literature.

## Background

Dear Editor,

We thank E. Colman & E. O’Connor for their article, “The role of WhatsApp® in medical education; a scoping review and instructional design model”, that clearly outlined the numerous benefits of IMAs as effective educational tools to drive learning. Instant messaging applications (IMAs) is an umbrella term encompassing many different online platforms, such as WhatsApp®, Facebook®, Twitter® and WeChat®, that provide a similar core service of rapid real-time message transmission between users via the internet. Focusing specifically on WhatsApp®, the review highlights that although it was not designed exclusively for educational purposes, its versatile nature offers unique opportunities to facilitate learning by providing a virtual platform for activities such as: group collaboration, peer communication and multimedia message sharing [1]. As senior medical students from St George’s University of London, reflecting upon our utilisation and level of dependence on WhatsApp in our daily student lives, we are in full agreement with regards to the findings this article brings forward. In this response, we further explore the benefits that WhatsApp has in improving the educational experience for medical students, specifically during problem-based learning (PBL) sessions and clinical attachments, whilst also considering how the potential integration of IMAs into the clinical setting can enhance the future delivery of healthcare.

## Main text

Since the turn of the century, smartphones have become increasingly prevalent and subsequently social media applications, such as IMAs, have infiltrated many dimensions of society, forming the backbone of communication. WhatsApp is the one of the most popular IMAs worldwide and is universal among medical students, owing to its affordable free-of-charge service and easy accessibility, with the application’s only prerequisites being a smartphone and an active internet connection [2]. It is therefore unsurprising that for medical students, WhatsApp® has overtaken email as the preferred mode of communication for establishing both social and academic peer-to-peer relationships, as email lacks the efficiency, convenience and “immediate” nature of IMAs. With the increasing popularity of IMAs coupled with the clear willingness of students to apply them in an educational manner, there is a definite case for tutors to promote the use of such technology in a teaching and learning environment [3].

### Problem based learning

The best example of peer-to-peer interactions over WhatsApp can often be seen within a PBL group setting, where typically a group chat will be created early on in the curriculum. These group chats extend interactive classroom discussions and facilitate the sharing of documents, links and images, thereby encouraging a collaborative approach to learning [3]. E. Colman & E. O’Connor document how WhatsApp allows the user to access relevant resources at their convenience, whilst raising questions and receiving information outside working hours, without having to arrange in-person meetings [1]. This is why instant messaging as a tool, when utilised for academic purposes, can improve student interaction, expand opportunities for student participation and stimulate learning through conversations rather than lectures; a notion that is central to PBL [3]. For tutors, the retrievability of exchanges, within the group chat, can be used as means of better assessing their students’ participation rates in discussions [4]. WhatsApp’s capacity to support audio group calls does offer a potential platform to host PBL sessions on in the future, however more research is required into the effectiveness of adopting this aspect of IMAs in a PBL setting and if the absence of face-to-face communication adversely impacts the quality of discussions.

### Clinical attachments

In clinical attachments, the lack of communication with clinical teaching fellows (CTFs) and other clinical supervisors presents unique challenges for medical students. Broadly speaking, clinical education supplied to students is often disorganised and based on a model of opportunistic teaching, where topics are usually determined through the conditions presented by patients and the preferences of tutors [2]. This is further complicated by the lack of communication channels medical students possess with their supervisors and vice versa, leading to little consistency in the delivery of teaching and sometimes a sub-optimal clinical experience.

The use of WhatsApp messaging, between students and their supervisors, goes some way to resolving these shortcomings of the system. E. Colman & E. O’Connor reviewed literature that illustrated WhatsApp’s ability to foster flexible and ad hoc style teaching in the absence of a pre-defined syllabus [1], a theme which is very applicable to the nature of clinical attachments. Instant messaging is used by tutors as a means of offering support, informing of timetable alterations and arranging impromptu bedside teaching sessions, if and when intriguing patient cases present themselves in order that students can fully utilise learning opportunities whenever they arise in the clinical setting [1, 2]. Implementing this type of communication provides a more structured placement experience, diminishes hierarchy and establishes better student-tutor relationships in an educational setting, leading to an overall more satisfying learning experience [2, 3]. Having said this, we believe that sharing patient information over WhatsApp should be avoided whenever possible, due to ethical concerns surrounding patient confidentiality. If circumstances do not permit this and exchanging such information is necessary for educational purposes, then both parties must exercise caution by making sure all information shared over WhatsApp is anonymised and can not be traced back to the patient.

The COVID-19 outbreak has severely impacted the delivery of the pre-registration medical curricula. In effort by hospitals to limit viral transmission, ease personal protective equipment shortages and shift CTFs towards patient care, there’s limited opportunity for inpatient and outpatient exposure for medical students in their clinical years. This state of affairs required teachers and students alike to acclimatise to learning at a distance whilst maintaining a level of normalcy. These unique set of challenges lead to the formal implementation of readily accessible technologies, such as video conference software and IMAs (Inc. WhatsApp), in delivering the core medical curricula by many institutions. A recent review evaluating the effectiveness of this emerging educational paradigm in an uncertain COVID-19 era, concluded that “virtual tools can be used by both learners and educators to achieve a shared goal of providing effective and efficient medical education”, but also adds that they are not without their inherent limitations [5]. Consequently, further research is needed to determine whether IMAs and other virtual platforms are an effective long-term substitute for in-person learning or if they should simply remain an adjunct to the traditional educational paradigm.

### Future of IMAs within the NHS

In recent decades, technological innovations in diagnostic equipment and treatment options have revolutionised patient care, yet communication systems within the healthcare setting have lagged behind and are now outdated. For the majority of the NHS hospitals in the United Kingdom (UK), the obsolete pager systems still form the foundation for clinical communication between healthcare professionals (HCPs). This technology is widely regarded as ineffective for communication, due to its high operating costs, disruptive nature, prolonged waiting times and inability to inform the recipient regarding the location or identity of the caller [2, 6]. This is very concerning as studies have shown that communication failures jeopardise patient safety, leading to detrimental consequences; these failures are commonly attributed to insufficient information transfer between HCPs [7].

WhatsApp has shown, in a very short period of time, to improve patient safety by overcoming and fixing the apparent flaws of the old communication systems [6], whilst having several added benefits of its own for HCPs as shown by E. Colman & E. O’Connor [1]. It aids clinical decision making, supports multimedia sharing between clinicians and provides documentation of practice, that can be further utilised for audit and training purposes [6]. WhatsApp’s nature of asynchronous communication allows the description and perceived urgency of the task to be displayed within the initial message, thereby granting the recipient the option to judge whether the incoming task justifies interruption of their current responsibilities. Unnecessary interruptions, as seen in the pager driven hospital environment, are known to incur extra costs in staff time and efficiency as well as having associated psychological factors, such as diversion of attention, forgetfulness and human errors [8]. In addition to this, team group chats foster an environment whereby a senior clinician can monitor jobs undertaken by their juniors without active interference, thus granting juniors a degree of clinical autonomy to enhance their learning [6].

With the rapid emergence of the COVID-19 global pandemic, the impact of which is being felt foremost on the front-line hospital wards means HCPs must operate with the upmost caution and co-ordination in providing treatment to patients. A recent study, retrospectively analysing conversations in a WhatsApp group chat between senior oncologists, concluded the usefulness of the platform in rapidly implementing new COVID-19 health measures for the management of cancer patients - a group especially at risk of life-threatening complications secondary to infection [9].

The introduction of end-to-end encrypted messaging has settled some concerns surrounding patient confidentiality and data privacy, which critics historically levelled against the platform [10]. Regardless, issues around data ethics still loom. For example, when clinical images received by WhatsApp are downloaded to the phone’s memory by default, often they are uploaded to a cloud-backup. From here, these images are accessible not only via other devices, but also from other countries [11]. Additionally, the practical element of utilising WhatsApp as route of easier communication within hospitals must be probed. An instance in which patient confidentiality could be undermined is where a HCP’s phone falls into the possession of someone with illegitimate interests. As WhatsApp does not require password entry, it is relatively straightforward therefore to access patient data at your fingertips [11]. Understandably, this leaves room for scrutiny; yet these faults are amendable through the development of an IMA specific for such needs.

The current healthcare secretary, M. Hancock Member of Parliament (MP), has ordered pagers to be phased out of NHS trusts completely by the end of 2021, with hospitals expected to replace them with modern 2-way communication systems prior to this deadline. West Suffolk NHS Foundation Trust underwent a pilot scheme in 2017, analysing the impacts of integrating a purpose-built instant messaging and calling system, Medic Bleep, into its working environment [12]. The capabilities of Medic Bleep, a service similar to WhatsApp, were primarily applied to making the inpatient discharge process more streamline, with a view of using it in other domains of care [13]. It was shown to have immediate impact on increasing productivity, saving junior doctors an average of 48 min per shift [9]. In the future, allowing medical students to participate in adopting similar innovations during their final years of study, could alleviate the current flaws that are all too prevalent in the delivery of clinical education. Additionally, it would result in newly qualified doctors being heavily familiar with such applications, like Medic Bleep, prior to starting practice thus negating the need for any extra training and ensuring a seamless transition.

## Conclusion

The potential IMAs have to enhance the delivery of the pre-registration medical curriculum has been well recognised for a number of years. WhatsApp’s ever-growing popularity and irreplaceability, among medical students and tutors, to facilitate learning from the classroom setting of PBL sessions to the erratic environment of clinical attachments, is a testament to this. This major shift in perception, from the days where phones were viewed solely as an interference, is down to the increased awareness of the opportunities IMAs have to offer for both students and tutors. The formal integration of IMAs into the healthcare setting has been sluggish over the last decade, despite the pressing need for the obsolete pager system to be overhauled. The UK government have now accelerated this process and promised the gradual implementation of purpose-built, two-way communication platforms such as Medic Bleep, in the coming years.

In our opinion, IMAs can serve as means of improving the way medical education is delivered, by creating communication channels between students and tutors, increasing learning opportunities and providing an overall superior educational experience for medical students. Our analysis of how NHS performance could be enhanced via IMA adoption is also evidence that other health systems internationally can benefit from such technology. However, as E. Colman & E. O’Connor concluded in their article, further research is required into the matter, especially concerning patient ethics [1]. The COVID-19 pandemic has presented an unforeseen opportunity to trial and thoroughly study the impact of such innovations in delivering medical education. Conclusively, introduction of IMA-type platforms into clinical practice will take time and patience though ad hoc updates in subsequent versions will bear fruitful for HCPs, patients and health systems worldwide.

## Data Availability

Not Applicable

## Abbreviations

NHS: National Health Service
IMA: Instant messaging application
PBL: Problem learning problem
CTF: Clinical teaching fellow
UK: United Kingdom
HCP: Health care professional
MP: Member of Parliament
